# Combination of gabapentin and ramosetron for the prevention of postoperative nausea and vomiting after gynecologic laparoscopic surgery: a prospective randomized comparative study

**DOI:** 10.1186/s12871-017-0357-8

**Published:** 2017-05-19

**Authors:** Kyung Mi Kim, Jin Huh, Soo Kyung Lee, Eun Young Park, Jung Min Lee, Hyo Ju Kim

**Affiliations:** 1 Department of Anesthesiology and Pain Medicine, Hallym University Sacred Heart Hospital, Hallym University College of Medicine, 22 Gwanpyeong-ro, 170 beon-gil, Dongan-gu, Anyang 431-796 Republic of Korea; 2 Department of Anesthesiology and Pain Medicine, Kangwon National University Hospital, Kangwon National University School of Medicine, 156 Baengnyeong-Ro, Chuncheon, Gangwon-Do 200-722 Republic of Korea

**Keywords:** Gabapentin, Ramosetron, Postoperative nausea and vomiting (PONV), Laparoscopic gynecologic surgery

## Abstract

**Background:**

As a drug originally introduced for its anticonvulsant effects, gabapentin has been recently shown to be effective in the treatment of nausea and vomiting in various clinical settings. This study compared the antiemetic efficacy of oral gabapentin, intravenous ramosetron and gabapentin plus ramosetron in patients receiving fentanyl-based patient-controlled analgesia after laparoscopic gynecologic surgery.

**Methods:**

One hundred and thirty two patients undergoing laparoscopic gynecologic surgery under general anesthesia were allocated randomly into three groups: group G received 300 mg oral gabapentin 1 h before anesthesia, group R received 0.3 mg intravenous ramosetron at the end of surgery, and group GR received a combination of 300 mg oral gabapentin 1 h before anesthesia and 0.3 mg intravenous ramosetron at the end of surgery. Postoperative nausea, retching, vomiting, rescue antiemetic drug use, pain, rescue analgesic requirements and adverse effects were assessed at 0–2, 2–24 and 24–48 h after surgery. Postoperative nausea and vomiting (PONV) was defined as the presence of nausea, retching or vomiting.

**Results:**

The incidence of complete response (no PONV and no rescue antiemetics up to 48 h postoperatively) was significantly higher in group GR (26/40, 65%) than group G (16/40, 40%; *P* = 0.025) and group R (18/44, 41%; *P* = 0.027), whereas there was no significant difference between group G and group R (*P* = 0.932). There were no significant between-group differences in the incidence of emetic episodes, use of rescue antiemetics, severe emesis, use of rescue analgesics or any adverse effects. Postoperative pain scores were also similar among groups.

**Conclusions:**

The combination with gabapentin and ramosetron is superior to either drug alone for prevention of PONV after laparoscopic gynecologic surgery.

**Trial registration:**

ClinicalTrials.gov NCT02617121, registered November 25, 2015.

## Background

Postoperative nausea and vomiting (PONV) is a frequent complication after general anesthesia, with an overall incidence of 40–90% [1]. Although PONV is generally self-limited, it can cause rare but serious medical complications, such as aspiration of gastric contents, suture dehiscence, esophageal rupture, subcutaneous emphysema or pneumothorax [2], all of which can significantly increase overall health care costs [3, 4].

Laparoscopic gynecologic surgery has been reported to be associated with a high incidence of PONV: approximately 80% in the absence of prophylactic antiemetics [5]. Thus, various pharmacologic agents, such as anticholinergics, antihistamines, promethazine, aprepitant, corticosteroids and 5-hydroxytryptamine (5-HT_3_) receptor antagonists, have been used to prevent and treat PONV in patients undergoing gynecologic laparoscopy [6–9]. However, PONV still occurs and its incidence reaches approximately 40% despite prophylaxis with a 5-HT_3_ receptor antagonist in patients with fentanyl-based intravenous (i.v.) patient-controlled analgesia (PCA) after gynecologic surgery [10]. Consequently, multimodal approaches consisting of two or more antiemetic therapies with different mechanisms of action have been highly recommended to reduce PONV in high-risk patients [11, 12].

In current practice, selective 5-HT_3_ receptor antagonists have been widely used as first- and second-line prophylaxis for preventing PONV because of their efficacy and relatively few side effects [12]. As a recently introduced selective 5-HT_3_ receptor antagonist, ramosetron has shown more potent and longer antiemetic effects than previous 5-HT_3_ receptor antagonists because of its strong binding affinity for, as well as slower dissociation rate from, 5-HT_3_ receptors [13]. A review article reported that the incidence of PONV during the first 0–24 h after anesthesia was lower in patients receiving ramosetron than in those receiving placebo after gynecologic surgery [14]. Several previous trials demonstrated that prophylactic ramosetron exhibits better efficacy than ondansetron in reducing PONV [15, 16].

As a drug originally developed for its anticonvulsant effects, gabapentin has been shown to be effective in the treatment of neuropathic and chronic pain [17]. The drug has also been extensively studied as a non-opioid alternative to decrease morphine requirements as part of a multimodal approach to managing postoperative pain [18]. Although the exact mechanism of action of gabapentin is not well understood, several studies have recently demonstrated gabapentin’s anti-nauseant effects in various clinical settings [19–21]. The administration of prophylactic gabapentin 600 mg orally reduced the incidence of PONV and antiemetic drug requirements after abdominal hysterectomy [22]. A study by Heidari et al. [23] comparing the effects of premedication with i.v. granisetron and oral gabapentin on the incidence and severity of PONV after middle ear surgery reported that the efficacy of gabapentin 300 mg in preventing PONV was similar to that of granisetron 3 mg up to 24 h after anesthesia. Another study found that the combination of preoperative oral gabapentin and i.v. dexamethasone before gynecologic procedures was associated with a lower incidence of PONV than that observed when each drug was administered separately [24].

Nevertheless, a comparison of the PONV-preventive effects of ramosetron versus gabapentin has not been heretofore performed. Moreover, it is unknown whether the concomitant use of these two drugs with different mechanisms is more effective than the use of each drug alone in patients at high risk of PONV. We therefore performed a prospective study to compare the efficacy of oral gabapentin versus i.v. ramosetron for PONV prophylaxis in patients with fentanyl-based i.v. PCA following gynecologic laparoscopy. In addition, we also evaluated whether combination prophylaxis with both drugs provides additional clinical benefit over that observed during monotherapy using just one of the drugs.

## Methods

### Study population

This prospective randomized study enrolled female patients who were classified as American Society of Anesthesiologists physical status I or II, aged 19–64 years and scheduled for therapeutic laparoscopic gynecologic surgery under general anesthesia at the Department of Obstetrics and Gynecology, Hallym University Sacred Heart Hospital, College of Medicine, Hallym University. All patients were anticipated to receive opioid-based i.v. PCA for postoperative pain management.

Patients were excluded for any of the following reasons: pregnancy or breastfeeding; psychological or psychiatric disease; administration of antiemetic medication or systemic corticosteroids within 24 h before surgery; vomiting within 24 h before surgery; alcohol or drug abuse; or known hypersensitivity or contra-indications to any of the drugs used in this study.

This prospective study was approved by the Institutional Review Board of Hallym University Sacred Heart Hospital (reference numbers: IORG0004993, IRB00005964). Written informed consent was obtained from each study patient before the administration of any study drugs. This trial was registered at ClinicalTrials.gov (NCT02617121).

### Study design and anesthesia protocol

Patients were randomly allocated into one of three groups (G, R or GR) using a computer-generated randomization method. Patients in group G received oral gabapentin (Gabapentin Cap®, Korea Drug Co., Seoul, Republic of Korea) 300 mg with small sips of water 1 h before induction of anesthesia and i.v. saline 2 mL at the end of surgery; patients in group R received i.v. ramosetron 0.3 mg in a total volume of 2 mL (Nasea®, Astellas Pharma Korea Inc., Seoul, Republic of Korea) at the end of surgery; and patients in group GR received a combination of oral gabapentin 300 mg 1 h before induction of anesthesia and i.v. ramosetron 0.3 mg in a total volume of 2 mL at the end of surgery. The study drugs were administered by a physician who did not participate in data collection.

All patients were allowed to take solid food up to 8 h before surgery and water 2 h before surgery. Glycopyrrolate 0.004 mg/kg was administered intramuscularly as premedication. When patients arrived in the operating room, standard monitoring, including limb lead electrocardiography, pulse oximetry, non-invasive blood pressure measurements, end-tidal anesthetic gas concentrations, capnography (CARESCAPE Monitor B650; GE Healthcare, Helsinki, Finland) and bispectral index monitor (BIS VISTA^TM^ Monitoring System; Aspect Medical Systems, Norwood, MA, USA), were applied. Induction of anesthesia was conducted with i.v. thiopental sodium 5 mg/kg, rocuronium bromide 0.6 mg/kg, and remifentanil 0.15–0.3 μg/kg/min. After 3 min of mask ventilation with 100% oxygen, endotracheal intubation was performed. Anesthesia was maintained with 1.5–3% sevoflurane, a continuous infusion of remifentanil 0.05–0.2 μg/kg/min, and air in oxygen (fraction of inspired oxygen, 0.5). The infusion rate of remifentanil and concentration of sevoflurane were adjusted to maintain the blood pressure and heart rate within 20% above or below baseline values and the BIS value between 40 and 60. Mechanical ventilation was adjusted to maintain an end-tidal carbon dioxide partial pressure of 35–40 mmHg throughout the procedure.

Approximately 30 min before the end of surgery, a bolus of i.v. fentanyl 1 μg/kg was given for postoperative pain control. At the end of the procedure, the sevoflurane and remifentanil were discontinued and neuromuscular blockade was antagonized using a combination of i.v. neostigmine 0.04 mg/kg and glycopyrrolate 0.008 mg/kg. The patients were extubated when fully awake. An i.v. PCA consisting of fentanyl 16–17 μg/kg and 0.9% saline in a total volume of 100 mL was provided for the first 48 h after surgery at a basal rate of 2 mL/h, bolus dose of 0.5 mL, and lockout time of 15 min. All patients also received i.v. ketorolac tromethamine (Keromin®, Hana Pharm Co., Seoul, Republic of Korea) 30 mg regularly every 8 h until 48 h after anesthesia for postoperative analgesia.

### Study assessments

Demographic data, Apfel’s risk score for PONV (consisting of female gender, nonsmoking status, history of PONV and/or motion sickness, and postoperative opioid use, with 1 point for each positive item) [5], duration of anesthesia, and duration of surgery were recorded for each patient. All episodes of PONV (nausea, retching or vomiting) were recorded during the first 48 h after anesthesia for three time periods: 0–2, 2–24 and 24–48 h. Nausea was defined as a subjectively disagreeable sensation accompanying the urge to vomit, retching was defined as rhythmic and spastic contractions of the respiratory muscles without ejecting gastric contents, and vomiting was defined as the forceful ejection of gastric contents from the mouth.

The primary outcome of this study was the incidence of a complete response within the first 48 h after anesthesia. Complete response was defined as the absence of PONV and lack of a need for rescue antiemetic therapy. Secondary outcomes were the incidence of severe nausea, emetic episodes and need for rescue antiemetics. Emetic episodes were defined as retching or vomiting. The severity of nausea was assessed according to an 11-point verbal numerical rating scale (VNRS, 0–10; 0 = no nausea, 10 = worst nausea imaginable) and classified as mild (1–3), moderate (4–6) or severe (7–10). These assessments were performed at the same times as the episodes of PONV assessments.

The rescue antiemetic, i.v. metoclopramide 10 mg, was administered for severe nausea or two or more emetic episodes, or upon a request from the patient. If PONV persisted after metoclopramide administration, i.v. ondansetron 4 mg was given. For patients who continued to have nausea after both metoclopramide and ondansetron, PCA was stopped for 2 h and the patients were observed. The number of administrations of rescue antiemetic drugs were recorded.

During the 48-h postoperative study period, patients were asked to rate their intensity of pain using an 11-point VNRS similar to that used for nausea. An i.v. bolus dose of 30 mg of ketorolac was administered upon request from the patient or when the VNRS pain score was ≥ 6. The number of rescue analgesic administrations was recorded. Data regarding adverse effects, such as dizziness, headache and drowsiness, was also collected. Postoperative sedation scores were evaluated using the following scale: 0 = awake, 1 = mild sedation, 2 = sleepy but arousable, and 3 = very sleepy. These assessments were performed at the same times as the episodes of PONV assessments. All data were recorded by an independent anesthesiologist who was blinded to the patient’s group assignment.

### Statistical analyses

The sample size calculation was based on the results of previously published studies in similar surgical populations and was performed using power analysis (α = 0.05, β = 0.8) to detect a 30% increase in the rate of a complete response. It indicated that 40 patients per group were required [25]. Assuming a potential dropout rate of 10%, the final sample size was set at 44 patients per group. The data are expressed as mean ± SD or number (%) of patients.

For intergroup comparisons, the distribution of continuous variables was first assessed for normality using the Shapiro–Wilk test. Normally distributed data were presented as mean ± SD and analyzed by one-way analysis of variance. Non-normally distributed data were expressed as median (interquartile range) and analyzed using the Kruskal-Wallis test. Categorical variables were analyzed using the Chi-square test or Fisher’s exact test, as appropriate. All significant results were further analyzed with Scheffe’s *post hoc* test to detect the intergroup differences. For all analyses, *P*-values were corrected using the Bonferroni method. *P*-value < 0.05 was considered to indicate statistical significance. A univariable logistic regression analysis was conducted to determine the correlation of the complete response (primary outcome of present study) with type of antiemetics (gabapentin, ramosetron or combination of gabapentin and ramosetron) and other variables in Table 1. Afterwards, a multiple logistic regression analysis was conducted with the confounding variables with a *P* value < 0.1 in a univariate logistic regression analysis to find independent factors associated with complete response. SPSS software version 22.0 (IBM Corp., Armonk, NY, USA) was used to analyze the data.

**Table 1:** Patients’ characteristics and clinical data

Characteristic	Group G (*n* = 40)	Group R (*n* = 44)	Group GR (*n* = 40)	*P* value (overall)
Age (year)	44.5 ± 11.1	44.2 ± 9.7	43.2 ± 9.2	0.831
Height (cm)	157.3 ± 5.4	158.3 ± 6.7	157.8 ± 4.9	0.753
Weight (kg)	60.5 ± 9.7	59.7 ± 11.3	61.9 ± 10.1	0.620
PONV history	4 (10.0)	3 (6.8)	2 (5.0)	0.683
Motion sickness history	3 (7.5)	8 (18.2)	6 (15.0)	0.349
Nonsmoking status	37 (92.5)	39 (88.6)	36 (90.0)	0.833
Apfel’s risk score for PONV				0.465
3	5 (12.5)	10 (22.7)	8 (20.0)	
4	35 (87.5)	34 (77.3)	32 (80.0)	
Type of laparoscopic surgery				0.414
Total hysterectomy^a^	13 (32.5)	21 (47.7)	21 (52.5)	
Ovarian cystectomy	17 (42.5)	10 (22.7)	9 (22.5)	
Myomectomy	6 (15.0)	7 (15.9)	5 (12.5)	
Salpingo-oophorectomy	4 (10.0)	6 (13.6)	5 (12.5)	
Duration of surgery (min)	102.0 ± 58.8	101.1 ± 53.1	101.3 ± 44.0	0.997
Duration of anesthesia (min)	146.1 ± 61.0	141.9 ± 58.1	136.1 ± 46.1	0.721
ASA class I/II	24/16	25/19	21/19	0.794

Data presented as mean ± SD or n (%) of patients

Data were analyzed using ANOVA (continuous variables) or *χ* ^2^ test (incidence variables)

Group G, patients received oral gabapentin 300 mg 1 h before induction of anesthesia; Group R, patients received intravenous ramosetron 0.3 mg at the end of surgery; Group GR, patients received oral gabapentin 300 mg 1 h before induction of anesthesia and intravenous ramosetron 0.3 mg at the end of surgery; *PONV* postoperative nausea and vomiting; Apfel’s risk score consists of four predictors: nonsmoking, female, history of motion sickness and/or PONV, postoperative opioid; *ASA* American Society of Anesthesiologists’ physical status

^a^ In some patients, salpingo-oophorectomy was perforemed together

## Results

**Fig. 1:**
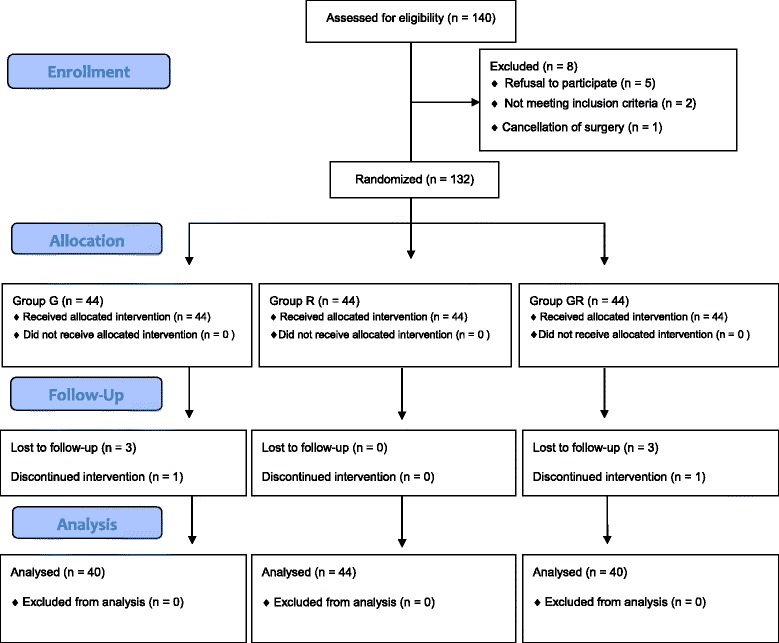
CONSORT diagram showing the flow of participants in present study

Among 140 patients enrolled in this study, 8 patients were excluded from the study due to following reasons: a refusal to participate (5 patients), not meeting inclusion criteria (2 patients) and a cancellation of surgery (1 patient). Hence, 132 patients were randomly allocated into three groups: group G (*n* = 44), group R (*n* = 44), and group GR (*n* = 44). Of these, 8 patients were withdrawn from the study: 6 patients because of incomplete data collection and 2 patients because propofol was used for anesthesia induction instead of thiopental. The final analysis included 44 patients in group R and 40 patients each in group G and group GR. A CONSORT flow diagram for the study is shown Fig. 1, and demographic and clinical data, including age, Apfel’s risk score for PONV, type of surgery, duration of surgery and duration of anesthesia, are presented in Table 1. No statistically significant between-group differences were found in any demographic or clinical characteristic (*P* > 0.05).

Data regarding the incidence of PONV, emetic episodes, rescue antiemetics and complete response during the 0–2, 2–24, 24–48 and 0–48 h postoperative periods are presented in Table 2. The proportion of patients without nausea was significantly higher in group GR than in group R and group G during the entire 48-h period after surgery (*P* < 0.05 for all comparisons). The number of complete responders (no PONV and no need for rescue antiemetics up to 48 h after surgery) was also higher in group GR (65%) than in group R (41%; *P* = 0.027) and group G (40%; *P* = 0.025), whereas no significant difference was found between group R and group G (*P* = 0.932). Group GR had a lower incidence of severe nausea, emetic episodes and rescue antiemetic use compared with group R and group G, but there were no statistically significant differences among the three groups (*P* > 0.05 for all comparisons) (Table 2).

**Table 2:** Incidence of postoperative nausea and vomiting, use of rescue emetics and complete response

Parameter	Group G (*n* = 40)	Group R (*n* = 44)	Group GR (*n* = 40)	*P* overall (*P*_1_, *P*_2_)
Postoperative 0–2 h				
Nausea (0/1/2/3)	22^*^/4/10/4	22^*^/6/4/8	32/2/1/5	0.042 (0.017, 0.038)
Emetic episode	4 (10.0%)	4 (9.1%)	4 (10.0%)	0.987
Rescue antiemetics	4 (10.0%)	5 (11.4%)	5 (12.5%)	0.939
Postoperative 2–24 h				
Nausea (0/1/2/3)	21^*^/7/8/4	22^*^/12/6/4	30/3/5/2	0.041 (0.036, 0.018)
Emetic episode	11 (27.5%)	10 (25.0%)	7 (17.5%)	0.564
Rescue antiemetics	5 (12.5%)	6 (13.6%)	4 (10.0%)	0.874
Postoperative 24–48 h				
Nausea (0/1/2/3)	29^*^/8/1/2	29^*^/9/4/2	36/3/1/0	0.030 (0.045, 0.008)
Emetic episode	2 (5.0%)	4 (9.1%)	1 (2.5%)	0.416
Rescue antiemetics	2 (5.0%)	2 (4.5%)	1 (2.5%)	0.831
Postoperative 0–48 h				
Severe nausea	9 (22.5%)	11 (25.0%)	6 (15.0%)	0.510
Emetic episode	14 (35.0%)	15 (34.1%)	9 (22.5%)	0.396
Rescue antiemetics	10 (25.0%)	9 (20.5%)	8 (20.0%)	0.834
Complete response	16 (40.0%)^*^	18 (40.9%)^*^	26 (65.0%)	0.038 (0.025, 0.027)

Data presented as n (%) of patients

Group G, patients received oral gabapentin 300 mg 1 h before induction of anesthesia; Group R, patients received intravenous ramosetron 0.3 mg at the end of surgery; Group GR, patients received oral gabapentin 300 mg 1 h before induction of anesthesia and intravenous ramosetron 0.3 mg at the end of surgery

Nausea: 0, none; 1, mild; 2, moderate; 3, severe; emetic episode: retching or vomiting; complete response: absence of postoperative nausea and vomiting and no need for rescue antiemetic therapy during the 48-h postoperative period

*P*_1_ = group G vs group GR; *P*_2_ = group R versus group GR

^*^ *P* < 0.05 compared with group GR

In the univariable logistic regression analysis, the type of antiemetic drugs (*P* = 0.042) and duration of anesthesia (*P* =0.077) were associated with the complete response. In the multiple logistic regression analysis, the type of antiemetic drugs was only significant predictor for complete response after adjusting for confounding factors. Patients who received gabapentin or ramosetron had a 0.377 or 0.371-fold lesser likelihood for complete response compared to those who received combination administration of gabapentin and ramosetron (95% confidence intervals, 0.148–0.928 and 0.154–0.923; *P* value = 0.034 and 0.033, respectively). But the duration of anesthesia was not associated with complete response after confounding adjustments (*P* = 0.099).

The rates of side effects, including dizziness, drowsiness and headache, were comparable among the three groups during the entire 48-h period. Additionally, there were no statistically significant differences among the three groups in VNRS pain scores or rescue analgesic requirements (Table 3). The sedation scores throughout the first 48 h after anesthesia were also not significantly different among the three groups (*P* > 0.05).

**Table 3:** Incidence of adverse effects, VNRS for pain and patients received rescue drug up to 48 h after anesthesia

Parameters	Group G (*n* = 40)	Group R (*n* = 44)	Group GR (*n* = 40)	*P* value (overall)
Adverse effects				
Dizziness	5	3	4	0.677
Headache	2	1	2	0.761
Drowsiness	1	0	1	0.572
VNRS for postoperative pain				
postoperative 0–2 h	6.4 ± 2.0	7.0 ± 2.0	6.3 ± 2.0	0.225
postoperative 2–24 h	3.2 ± 1.4	3.5 ± 1.5	3.3 ± 2.0	0.608
Postoperative 24–48 h	1.7 ± 1.0	1.9 ± 1.0	1.5 ± 1.4	0.180
Rescue analgesic requirements				
postoperative 0–2 h	8 (20.0%)	11 (25.0%)	9 (22.5%)	0.861
postoperative 2–24 h	0 (0.0%)	2 (4.5%)	0 (0.0%)	0.158
Postoperative 24–48 h	1 (2.5%)	1 (2.3%)	0 (0.0%)	0.614

Data presented as mean ± SD or n (%) of patients

Group G, patients received oral gabapentin 300 mg 1 h before induction of anesthesia; Group R, patients received intravenous ramosetron 0.3 mg at the end of surgery; Group GR, patients received oral gabapentin 300 mg 1 h before induction of anesthesia and intravenous ramosetron 0.3 mg at the end of surgery; *VNRS* verbal numerical rating scale 0–10; 0 = no nausea, 10 = worst nausea

## Discussion

In the present study, we found that oral gabapentin 300 mg administered 1 h before induction of anesthesia and i.v. ramosetron 0.3 mg given at the end of surgery were associated with comparable PONV-preventive effects in patients undergoing laparoscopic gynecologic surgery, a population at high risk for PONV. In addition, the combination of oral gabapentin 300 mg and i.v. ramosetron 0.3 mg was more effective in preventing PONV than gabapentin or ramosetron monotherapy.

Although the etiology of PONV has not been well elucidated, the occurrence of PONV depends on multiple factors, including the individual patient’s susceptibility, as well as anesthesia- and surgery-related factors [26]. Patient-specific risk factors for PONV in adults include female sex, a history of PONV and/or motion sickness, nonsmoking status and young age [27]. Additionally, volatile anesthetics, nitrous oxide and intra- and postoperative opioid use such as i.v. PCA with fentanyl or morphine are strongly regarded as the most likely causes of PONV in many instances. Laparoscopic surgery, gynecologic surgery and cholecystectomy have all been identified as independent risk factors for PONV [28]. Most participants in the current study had several of the abovementioned risk factors, including female sex, nonsmoking status, laparoscopic surgery, volatile anesthetic use and intra- and postoperative opioid use, thus suppression of PONV was an important issue for these patients.

Many studies have been conducted to find ways to reduce the incidence and severity of PONV. In current practice, various pharmacotherapies, including 5-HT_3_, dopaminergic, histaminic and NK_1_ antagonists, have been used for antiemetic prophylaxis. Few trials have compared known antiemetics and gabapentin with regard to their efficacy in reducing PONV. Jahromi et al. [29] found that oral premedication with chloropromazine 25 mg, gabapentin 300 mg, and metoclopramide 10 mg before maxillofacial trauma surgery could lead to a significantly reduced incidence of PONV. A report from Heidari et al. [23] of patients undergoing middle ear surgery indicated that preoperative oral gabapentin 300 mg and i.v. granisetron 3 mg had similar effects in decreasing the incidence and severity of PONV.

The present study is the first to compare ramosetron and gabapentin as antiemetic agents. Although a variety of 5-HT_3_ receptor antagonists have been shown to be effective in preventing PONV on the basis of their efficacy and safety following gynecologic laparoscopic surgery [7], the higher costs of 5-HT_3_ receptor antagonists remain a major drawback. Moreover, co-administration of antiemetics with different mechanisms of action has been suggested as a way to increase the complete response rate of patients who have an elevated risk of PONV [11, 12]. In general, 5-HT_3_ receptor antagonists plus various drugs from different classes, including i.v. dexamethasone 4–5 mg, i.v. droperidol 0.625–1.25 mg, and oral aprepitant 40 mg have been shown to reduce PONV to a greater extent than single therapy with any of the drugs [6, 30–32]. These results are consistent with our study. In our trial, addition of gabapentin to ramosetron led to a further reduction in the incidence PONV (to 24%), without the appearance of substantial side effects. Furthermore, gabapentin is a relatively inexpensive medication, the use of which can result in significant cost savings [20]. Therefore, gabapentin may be a useful choice for combination therapy to prevent PONV, especially in high-risk patients. Consequently, on the basis of its safety profile and cost, gabapentin might be beneficially included in the current list of PONV prophylactic regimens.

The actual mechanism by which gabapentin suppresses nausea and vomiting has been under discussion. It has been suggested that gabapentin reduces calcium signaling in the area postrema [33]; mitigates tachykinin neurotransmitter activity [34]; decreases perioperative inflammation at the site of surgical trauma, resulting in decreased postoperative ileus and subsequent PONV [35]; and reduces perioperative opioid requirements [36]. It seems reasonable to conclude that some combination of the abovementioned mechanisms may be responsible for gabapentin’s antiemetic efficacy.

In this study, it is interesting that the proportion of patients without nausea at 24–48 h after anesthesia was high in the group GR regarding elimination half life (4.8–8.7 h) and duration (8–12 h) of action of gabapentin [29, 37]. This is clinically meaningful when considering the report that the PONV symptoms can appear up to at least 72 h after discharge from PACU [38]. This finding might be explained in part by the possibility of long-lasting (> possibly 24 h) antiemetic effect of gabapentin [2]. Further clinical trials are required to address this issue.

A previous study by Achuthan et al. [2] of the use of gabapentin in patients undergoing abdominal surgery demonstrated the differential antiemetic efficacy of gabapentin with respect to the use of propofol, either as an induction or maintenance agent. Preoperative gabapentin as pharmacotherapy for preventing PONV was effective when propofol was not used. They suggested the anti-emetic effect of propofol may be the explanation of the differential effects of gabapentin. One of the goals in our study was the evaluation of the PONV-preventive effects gabapentin in comparison with ramosetron. Therefore, we used thiopental and sevoflurane as induction and maintenance agents instead of propofol. Further studies comparing the efficacy of gabapentin with ramosetron with the use of propofol are needed to get the more precise information about the role of preoperative gabapentin in PONV.

In the present study, the postoperative pain scores and use of rescue analgesic were not different among all groups whether they did or did not receive gabapentin. These findings were contrary to the results of several previous studies demonstrating that oral gabapentin 400 or 600 mg was effective in decreasing the postoperative pain and opioid consumption in patients undergoing surgery. [22, 24]. One possible explanation for these discrepancies is that different dosage of gabapentin used in these studies.

Although our study demonstrated that oral gabapentin and i.v. ramosetron showed comparable antiemetic effect after laparoscopic gynecologic surgery under general anesthesia, it should be cautiously interpreted that both drugs have pharmacologically equal effects as antiemetic agents for PONV prophylaxis due to the following reasons. First, a control group was not included in the present study because of ethical reasons, thus direct comparisons with no treatment were not conducted. Also, according to different routes of administration (oral route in gabapentin vs. intravenous route in ramosetron), duration of NPO which may affected the occurrence of PONV was not equal in participants received gabapentin and ramosetron because small sips of water 1 h before induction of anesthesia were only allowed in patients received oral gabapentin. Finally, previous studies demonstrated that preoperative gabapentin significantly reduce postoperative opioid requirements [22, 24]. In the present study, elastometric balloon infuser, which is the clinical postoperative pain management protocol we use routinely in our practice for gynecologic surgeries, were used as the PCA device not electronic infuser. We therefore could not assessed fentanyl consumption via i.v. PCA at each time period. Thus, it is not obvious that the decreased PONV incidence was associated with gabapentin’s antiemetic property or reduction in postoperative opioid consumption. The recent report documented the gabapentin’s antiemetic effect would be mediated by the mechanisms other than the decreased consumption of opioids [2]. Future studies are needed to precisely define the mechanism of antiemetic effect of gabapentin.

In general, the use of PONV prophyl agents is accompanied by the risk of various adverse events. In case of ramo, side effects ranging in severity from mild headache, drowsiness, dizziness, numbness of tongue, redness and diarrhea to potentially significant QTc prolongation, which may infrequently cause unexpected cardiac arrest may occur [39]. A recent meta-analysis of patients receiving gabapentin as antiemetic prophylaxis found that gabapentin in doses of 300, 600 and 900 mg produced no statistically significant sedation, whereas preoperative administration of gabapentin 1200 mg was associated with significantly greater postoperative sedation than controls [21]. In the present study, no significant differences in postoperative sedation were observed among all groups, whether they did or did not receive gabapentin. Moreover, in the current study, combining gabapentin and ramosetron did not increase the incidence of adverse events, such as dizziness, drowsiness and headache.

The current study had additional limitations. First, oral placebo drugs were not administrated in the ramosetron group, which may have affected blinding. Secondly, we tested the efficacy of only one dosage of gabapentin and ramosetron. It is, therefore, not known whether different dosages of either or both drugs would have produced similar antiemetic efficacy. Further clinical investigations to determine the effects of diverse dosages of the two drugs as prophylactic regimens for preventing PONV should be performed in the future. Thirdly, we did not carry out a thorough investigation into the history of preoperative use of drugs which exert influence on the occurrence of PONV, such as antacid or prokinetic drugs.

## Conclusions

In conclusion, the combination of gabapentin and ramosetron provided additional beneficial effects over ramosetron or gabapentin alone for high-risk patients requiring combination antiemetic prophylaxis. Based on the safety profile, known analgesic properties and cost, gabapentin might be usefully included in the list of pharmacotherapies for PONV prophylaxis in patients undergoing gynecologic laparoscopic surgery.

## Abbreviations

5 HT_3_: 5-Hydroxytryptamine 3
BIS: Bispectral index
PCA: Patient-controlled analgesia
PONV: Postoperative nausea and vomiting
SD: Standard deviation
VNRS: Verbal numerical rating scale
